# Reinforcement Learning and Dopamine in Schizophrenia: Dimensions of Symptoms or Specific Features of a Disease Group?

**DOI:** 10.3389/fpsyt.2013.00172

**Published:** 2013-12-23

**Authors:** Lorenz Deserno, Rebecca Boehme, Andreas Heinz, Florian Schlagenhauf

**Affiliations:** ^1^Max Planck Institute for Human Cognitive and Brain Sciences, Leipzig, Germany; ^2^Department of Psychiatry and Psychotherapy, Charité – Universitätsmedizin Berlin, Berlin, Germany

**Keywords:** schizophrenia, dopamine, computational modeling, reinforcement learning, aberrant salience, prediction error, fMRI, PET imaging

## Abstract

Abnormalities in reinforcement learning are a key finding in schizophrenia and have been proposed to be linked to elevated levels of dopamine neurotransmission. Behavioral deficits in reinforcement learning and their neural correlates may contribute to the formation of clinical characteristics of schizophrenia. The ability to form predictions about future outcomes is fundamental for environmental interactions and depends on neuronal teaching signals, like reward prediction errors. While aberrant prediction errors, that encode non-salient events as surprising, have been proposed to contribute to the formation of positive symptoms, a failure to build neural representations of decision values may result in negative symptoms. Here, we review behavioral and neuroimaging research in schizophrenia and focus on studies that implemented reinforcement learning models. In addition, we discuss studies that combined reinforcement learning with measures of dopamine. Thereby, we suggest how reinforcement learning abnormalities in schizophrenia may contribute to the formation of psychotic symptoms and may interact with cognitive deficits. These ideas point toward an interplay of more rigid versus flexible control over reinforcement learning. Pronounced deficits in the flexible or model-based domain may allow for a detailed characterization of well-established cognitive deficits in schizophrenia patients based on computational models of learning. Finally, we propose a framework based on the potentially crucial contribution of dopamine to dysfunctional reinforcement learning on the level of neural networks. Future research may strongly benefit from computational modeling but also requires further methodological improvement for clinical group studies. These research tools may help to improve our understanding of disease-specific mechanisms and may help to identify clinically relevant subgroups of the heterogeneous entity schizophrenia.

## Introduction and Outline

The “dopamine-hypothesis” of schizophrenia was initially built upon the observation that dopamine receptor antagonists, such as haloperidol, attenuate psychotic symptoms (1). Evidence showing that elevated dopamine levels are indeed involved in the pathophysiology of psychotic symptoms and schizophrenia is primarily derived from neurochemical studies using positron-emission-tomography (PET) with radioactive ligands targeting the brain’s dopamine system. Such studies clearly indicate elevated levels of presynaptic dopamine function (2, 3) with particularly strong evidence from meta-analyses for elevated dopamine synthesis capacity (4, 5). A hallmark of dopamine research was the observation that phasic releases of dopaminergic neurons code a temporal-difference prediction error, which was later shown to be causally involved in learning (6–8). This ability to form predictions about future outcomes is fundamental for interactions with the environment and depends on neuronal representations of such teaching signals. Behavioral impairments in reinforcement learning are a key finding in schizophrenia patients and have been proposed to be closely linked to reports of elevated presynaptic dopamine neurotransmission. Influential theoretical work suggests that dysfunctional reinforcement learning may contribute to the formation of the prominent clinical characteristics of schizophrenia patients, namely positive and negative symptoms (9–11). Furthermore, prediction errors are involved in learning-related changes in synaptic plasticity (12), and aberrant plasticity has been suggested as a potential common biological mechanism characterizing the schizophrenia spectrum (13, 14).

Embedded in this context, the central attempt of this article is to review studies on reinforcement learning in schizophrenia and to disentangle dimensions of symptom formation and potential disease-specific mechanisms in the existing literature. The primary focus of this article is to provide an up-to-date overview of the existing literature with the aim to review existing evidence for two influential theories. Therefore, we only include a brief introduction (see Reinforcement Learning in Schizophrenia: Theoretical Considerations) to these hypotheses and refer to the original publications for more detailed theoretical descriptions. The empirical studies reviewed here comprise behavioral and functional neuroimaging studies [restricted to functional magnetic resonance imaging (fMRI) and PET] in patients suffering from schizophrenia. In the first part, we start with studies on reward anticipation and processing based on pre-learned contingencies. Subsequently, we focus on studies that directly examine learning over time with a focus on studies that implemented reinforcement learning models. Finally, we summarize studies that combined experimental perturbations of the brain’s dopamine system, such as pharmacological challenges and molecular imaging (PET), with measures of reinforcement learning.

## Feedback Anticipation and Processing

A series of studies used the monetary incentive delay task (MID), a paradigm invented by Knutson and colleagues [(15), see also Ref. (16)]. The initial study demonstrated that participants speed up motor responses to obtain rewards and that anticipation as well as delivery of rewards evoke ventral striatal activation. The first application of this task in schizophrenia patients was carried out by Juckel and colleagues: they found reduced ventral striatal activation in unmedicated patients (17). This finding was subsequently replicated in a larger cohort of drug-naïve, first-episode patients (18, 19). In the study by Juckel et al. (17), it was demonstrated that blunting of anticipatory ventral striatal activation elicited by monetary reward reflected the individual degree of negative symptoms (17). This association was also present in patients treated with typical or first generation antipsychotics (FGAs, or “typical” antipsychotics), who showed reduced ventral striatal activation during reward anticipation, while patients treated with atypical or second generation antipsychotics (SGAs, or “atypical” antipsychotics) showed intact activation during anticipation of monetary reward in the same region (20, 21). This effect of SGAs was recently replicated in a larger cohort of patients (22). In line with these results, further studies applying the MID task in chronic schizophrenia patients medicated predominantly with SGAs did not find reduced ventral striatal anticipation of monetary reward in the patient group, as a whole (23–25). Two studies replicated the association of reward anticipation with negative symptoms (25) and apathy (24), while two other studies reported a correlation of ventral striatal activation during reward anticipation with positive symptoms (18, 19).

Although the static MID task is thought to mirror aspects of animal experiments studying reinforcement learning in the dopaminergic system [e.g., Ref. (6)], the gross time scale of fMRI compared to neurophysiological studies needs to be taken into account (26). Nevertheless, it has been demonstrated that ventral striatal activation during reward anticipation is indeed modulated by dopamine: a positive correlation between the anticipatory activation in core dopamine areas and reward-induced dopamine release was observed via competition of endogenous dopamine with a PET D2/3-receptor radioligand (27). In a study by Knutson et al. (28), diminished ventral striatal reward anticipation was reported when comparing healthy participants receiving amphetamine (resulting in a massive release of dopamine) to placebo (28). The latter study coincides with the results reported above in schizophrenia patients during reward anticipation and the well-established finding of elevated presynaptic dopamine function in schizophrenia using PET with FDOPA and similar tracers [for meta-analyses see: Ref. (4, 5)]. Based on this, it appears conceivable that event-related responses to reward-indicating cues disappear in the noise of elevated dopaminergic activity observed in schizophrenia patients and that this may ultimately contribute to a failure of salience attribution to environmentally relevant stimuli (9, 10, 29). Interestingly, Esslinger et al. (18) implemented the MID task in combination with another task possibly reflecting salience and showed in an exploratory correlation analysis that more pronounced ventral striatal hypoactivation during reward anticipation was associated with more salience attribution to neutral stimuli (18). In line with this, a recent study using emotional picture stimuli demonstrated that schizophrenia patients rate neutral pictures as more salient (30). These results provide some rather indirect support for the idea of aberrant salience in schizophrenia, which we will briefly introduce in the following section.

In contrast to reward anticipation, fewer studies used the MID task to examine the delivery of monetary outcome. One study (31) found that violations of outcome expectancies triggered abnormal neural responses in unmedicated patients: While medial-prefrontal activation was exaggerated when an expected-reward was omitted, ventral striatum (VS) displayed reduced activation for successful versus unsuccessful loss avoidance. The degree of delusion severity was found to be associated with activation in medial-prefrontal cortex (PFC) for successful versus unsuccessful loss avoidance. Moreover, functional connectivity between VS and medial PFC was reduced in patients. In a similar vein, Waltz et al. (25) found reduced activation in the medial PFC and lateral PFC when comparing win versus loss trials in schizophrenia patients medicated with SGAs. Activation to reward delivery in lateral PFC was negatively correlated with the degree of positive and negative symptoms (25). Another study (23) tested high and low rewards together with high and low punishments against neutral events and found significant activation in lateral PFC of healthy controls, most likely reflecting salience. This activation pattern was diminished in patients treated with SGAs. Interestingly, a recent study showed exaggerated activation in dorsolateral PFC elicited by neutral outcomes in unmedicated patients (19).

Two studies examined classical conditioning that actually took place outside the MRI scanner (32, 33). These designs might be thought of as extensions to studies using the MID task: Contingencies were pre-learned before scanning, but allow one to distinguish between expected-rewards, unexpected-rewards (presumably mirroring positive prediction errors), and unexpected omissions of rewards (presumably mirroring negative prediction errors; (32). Juice was used as a primary reinforcer in 18 medicated patients (32). Attenuated neural responses in dopaminergic core areas (midbrain and striatum) to expected and unexpected-reward deliveries were observed, while activation in reward omission trials was largely intact. Morris et al. (33) completed this approach in a full 2 × 2 design, thereby enabling an orthogonalization of the factors “rewards” and “surprise” as well as the interaction of both factors, which is assumed to mirror prediction-error-related brain activation. In 21 schizophrenia patients medicated with SGAs, this revealed a disrupted differentiation between expected and unexpected events in a way that ventral striatal activation is not coding prediction errors: while response to expected events in right VS was exaggerated, response to unexpected outcomes in left VS was found to be blunted (33).

In summary, fMRI studies in reward processing using the MID task have so far provided important insights into the neural processes underlying outcome anticipation and delivery in schizophrenia. In particular, the finding of reduced ventral striatal activation during outcome anticipation was consistently replicated across three studies involving a total of 68 unmedicated patients. An association of anticipatory ventral striatal activation with negative symptoms was reported in five studies involving 10 unmedicated patients and 52 medicated patients. Antipsychotic medication remains a crucial issue since these drugs specifically block those striatal D2-receptors that are (among others) activated by potentially prediction-error-associated dopamine release [e.g., Ref. (34, 35)] and moreover affect presynaptic dopamine synthesis (36, 37). Therefore, assessing unmedicated patients is key to understanding dopamine dysfunction in schizophrenia and to avoiding confounds by medication effects, which also appear to differ depending on FGAs versus SGAs (20, 21). Furthermore, one important limitation of the studies discussed thus far is the fact that all reward contingencies are pre-learned (i.e., before participants enter the MRI scanner and perform the task). Anticipatory brain activation during the MID task is likely to capture some aspects of reinforcement learning in particular with respect to cue- or action-related value signals. Some kind of value quantification is usually the main outcome variable of reinforcement learning models. It is important to note that these functions evolve over time, which is also a fundamental principle of brain signals. This points out an important limitation of the MID studies which may therefore provide a rather coarse proxy of value-related brain activation and consequently emphasizes the necessity to study learning over the course of time. Thus, studying the temporal dynamics underlying the actual learning process may provide more insights into symptom- and disease-specific processes associated with schizophrenia. In contrast to studies which used the MID task or similar designs, all studies discussed in Section “Behavioral Studies of Reinforcement Learning in Schizophrenia” refer to experimental paradigms that investigate learning on a trial-by-trial basis. Detailed computational modeling of such temporal dynamics may be particularly helpful to elucidate dysfunctional processes in patients and to improve characterization of a heterogeneous disease entity that is so far still based on symptoms (38–41).

## Reinforcement Learning in Schizophrenia: Theoretical Considerations

Reinforcement learning represents a promising, theory-driven tool (42) which aims to quantify learning on a trial-by-trial basis and has so far been implemented in a limited number of clinical group studies [e.g., Ref. (43), Table 1]. Although there are several different variants of models, most of them separate two main contributors to the learning process and both of them change on trial-by-trial (Box 1): first, the delivered outcome which refers to the time point when prediction errors arise. This teaching signal is thought to be crucially involved in driving any learning process. Second, the values of environmental cues or actions which are learned via this teaching signal. Concepts of motivational or incentive salience are closely linked with values of actions or environmental cues (44) that can be acquired during prediction-error-driven trial-and-error learning. Differences in the perceived properties of feedback stimuli *per se* (e.g., shifts in hedonic experience or salience) may also influence the elicitation of prediction errors and thus potentially corrupt learning processes. Based on these two main time points, we will proceed with a brief summary of two influential hypotheses with respect to the potential contribution of reinforcement learning to symptom dimensions and disease-specific features in schizophrenia.

**Table 1:** **Studies in schizophrenia patients using a computational model approach**.

Reference	Paradigm	Methods	Model	Main findings
Strauss et al. (89)	Temporal utility integration task	51 Medicated schizophrenia and schizoaffective patients, behavioral data only	RT-based RW	Impaired go, intact nogo learning in patients, correlation with negative symptom level
Gold et al. (64)	Instrumental probabilistic reward-approach versus punishment avoidance learning	47 Medicated schizophrenia and schizoaffective patients, behavioral data only	Actor-critic Q-learning hybrid of these two	High negative symptoms patients fail to represent and learn from reward value properly, loss avoidance is preserved
Murray et al. (43)	Instrumental reward learning	13 First-episode patients, 8 on SGAss, later diagnosed: 1 bipolar, 1 psychosis, 11 schizophrenia, fMRI	Q-learning	Impaired differentiation between neutral and reward predicting stimuli, attenuated activity for reward predicting stimulus, trend-wise augmented for neutral stimulus, reduced RPE activity in midbrain and VS
Koch et al. (103)	Instrumental gambling task	19 Medicated (except 1) schizophrenia patients, fMRI	TD	Impaired behavioral performance, reduced dorsolateral PFC and cingulate gyrus probability related activity, reduced RPE response in PFC, putamen, hippocampus and insula
Gradin et al. (106)	Instrumental probabilistic reward learning	15 Medicated schizophrenia patients, fMRI	SARSA-TD	Less rewards achieved, reduced RPE related activity in striatum, thalamus, amygdala-hippocampal complex, and insula, reduced encoding of expected value in amygdala-hippocampal complex and parahippocampal gyrus, correlation with positive symptoms
Romaniuk et al. (93)	Aversive classical conditioning	20 Medicated schizophrenia patients, fMRI	TD	No difference in RT, difference in skin conductance, impaired amygdala activation during conditioning, impaired midbrain activation during learning, inappropriate activation of nucleus accumbens in response to neutral cues
Schlagenhauf et al. (77)	Instrumental reversal learning task	24 Unmedicated schizophrenic patients, fMRI	RW, double-update, Hidden–Markov	Deficit in reversal learning, relation to positive symptoms, VS learning signals are reduced independent of task insight in contrast to prefrontal activation

*RW, Rescorla–Wagner-model; TD, temporal-difference model; SARSA, state action response state action; RPE, reward prediction error; VS, ventral striatum; RT, reaction time*.

Box 1 **Reinforcement learning models.**

A prediction error is defined as the difference between a delivered reward *R* and an expected value, here denoted as *Q*. *t* and *a* denote indices that refer to time and the value associated with a chosen action, respectively.

(1) $$\delta_{Qa,t}=R_{t}-Q_{a,t}$$

In model-free learning, this error signal can be used to update values:

(2) $$Q_{a,t+1}=Q_{a,t}+\alpha \delta_{Qa,t}$$

Here, α represent a learning rate, which weighs the influence of δ*_Qa,t_* on *Q_a,t_*_ + 1_ with natural boundaries between 0 and 1. For examples of clinical studies using this algorithm, please compare Murray et al. (43) or Schlagenhauf et al. (77). Equation 2 refers to environments, in which each time point or trial *t* consists of one stage, e.g., one action, which results in feedback delivery. This can be extended to sequential decision tasks, where each trial consists of multiple numbers of stages and for example only the final stage is associated with feedback delivery. For an extension of the Eqs 1 and 2 for sequential decisions, please compare the work by Daw et al. (80) or Glascher et al. (79).

Still referring to model-free learning, we can define δ and the update equation differently, as for example in actor-critic models. The same error signal, generated by the critic, updates values of the critic and the actor:

(3) $$\begin{array}{ll} \delta_{C,s,t} & =R_{t}-C_{s,t} \end{array}$$

(4) $$\begin{array}{ll} C_{s,t+1} & =C_{s,t}+\alpha_{s}\delta_{Cs,t} \end{array}$$

Notably, the critic Eqs 5 and 6 neglects the specific action that was chosen in trial *t*. The actor learns specific action values via the same error signal δ*_Cs,t_*:

(5) $$A_{s,a,t+1}=A_{s,a,t}+\alpha_{s}\delta_{Cs,t}$$

This approach was applied in one clinical study (64).

So far, all presented models are examples for model-free learning. Subsequently, we present one example, which touches the ground of model-based learning. Depending on task structure, it is possible to implement certain aspects of the environment. For instance, in an environment with two choice options prediction errors may also be used to update values of unchosen actions *ua*; this can be done by an additional extension of Eq. 2:

(6) $$Q_{ua,t+1}=Q_{ua,t}-\alpha \delta_{Qa,t}$$

Equation 8 represents a full double-update learner (77), while it is also possible to weigh the influence of the double-update by adding another free parameter:

(7) $$Q_{ua,t+1}=Q_{ua,t}-\kappa \alpha \delta_{Qa,t}$$

Here, we use chosen prediction errors to update unchosen values. Based on the task design, it may be possible to use unchosen prediction errors (143). An elegant approach is to mix values learned by two different algorithms. This can be achieved by introducing a weighing parameter, for example as in Eq. 7. Please note that the contribution of additional free parameters (e.g., different learning rates for rewards and punishments in Eq. 2 or different learning rates for the critic and the actor in Eqs 4 and 5) needs to be quantified and that this is ultimately a question answered by model selection procedures [e.g., Ref. (115)].

For all the described models, learned values need to be transformed into choice probabilities to generate behavior. One commonly used approach is the softmax equation, which can be written as:

(8) $$p\left(a,t\right)=\frac{\exp\left(\beta \times Q_{a,t}\right)}{\sum_{a\prime }\exp\left(\beta \times Q_{a\prime ,t}\right)}$$

Here, all models refer to instrumental tasks. Most of the equations are applicable in similar forms to classical conditioning. For detailed reading, we refer to the scholarly book by Sutton and Barto (42).

We begin with the “aberrant salience” hypothesis: schizophrenia patients may attribute salience to otherwise neutral environmental stimuli, and those stimuli may ultimately appear meaningful and evoke delusional mood in patients (9, 10). This process has been described as closely linked to a dysregulation of the dopamine system where both chaotic dopamine firing (45) and elevated baseline dopamine levels (46, 47) have been proposed to be involved. Whether this process actually reflects reinforcement learning in the same way as it was theoretically and mechanistically defined for healthy people (42) remains an open and exciting question. If this is the case, then neutral events should elicit prediction errors which may consequently train values for the associated cues or actions, and these values may finally exceed incentive values associated with rewarding or otherwise reinforcing events. In other words, patients are assumed to attribute importance to stimuli ignored by healthy volunteers and thereby learn something else. The degree of this alteration should be related to positive symptom levels, in particular delusions. It is important to note, that a prerequisite for the latter idea is that misattributed salience to certain neutral events remains stable over a period of time. Alternatively, it may also be possible that the process of misattributing salience is fluctuating permanently, resulting in a random pattern (a state where “everything is salient”) that would formally result in no learning at all and might therefore be harder to quantify. It is also conceivable that aberrant aspects of reinforcement learning have not yet been formulated correctly. Here, the role of unsigned prediction errors, as a valence-unspecific salience signal, might be of interest and could possibly be integrated in models of reinforcement learning (48–50).

The second hypothesis focuses on a deficit in the representation of learned values (11). This hypothesis posits that prediction errors are not adequately used to learn values even though hedonic experience itself remains mainly intact. This concept relates closely to the idea that reward feedback is not adequately transformed into motivational drive for goal-directed behavior (51) and has been proposed as a potential mechanism for the origin of negative symptoms (11). In general, a failure to learn any value may also be based on a reduction of hedonic experience, in which case no prediction errors are elicited and therefore no values can be learned; based on studies reviewed in the next section, this appears to be unlikely in schizophrenia patients. On the other hand, a deficit in using monetary and primary rewards for motivated behavior would appear similar to what was proposed in the incentive-sensitization theory of addiction disorders, which assumes a shift from non-drug rewards to drug-related rewards (44). In schizophrenia, such a shift may predominantly concern neutral stimuli and therefore result in aberrant learning as pointed out in the aberrant salience hypothesis.

As indicated, the two hypotheses are only partially independent. It is possible that both mentioned mechanisms exist in parallel and converge in producing a behavioral deficit but diverge in their differential contribution to symptom formation. In the following, we will review studies that aimed to test these hypotheses. Thereby, we try to build a coherent picture of how reinforcement learning may contribute to the formation of psychotic symptoms and if this appears to be dimensional or categorical. Finally, we endeavor to interpret previous studies with regard to their disease specificity by summarizing and discussing those studies that examined learning over time. We start with behavioral studies followed by a section on imaging studies. We also mention if studies implemented models of reinforcement learning and how parameters underlying these models were inferred.

## Behavioral Studies of Reinforcement Learning in Schizophrenia

Behavioral deficits in associative learning, particularly in instrumental tasks where feedback is used to guide behavior, are frequently replicated in schizophrenia patients. So far, only seven studies have implemented models of reinforcement learning (see Table 1), and although reinforcement learning modeling quantifies the observed behavior, only two of these studies were purely behavioral; the other five studies also collected fMRI data and regressed model-derived learning time-series (e.g., prediction errors) against imaging data. Studies on classical conditioning are reported in the subsequent section, because all the clinical studies conducted so far have assessed classical conditioning effects via physiological measures. In the following we will summarize studies that used instrumental tasks. We will also describe modeling studies in detail, because this approach represents a powerful tool to provide a more fine-grained understanding of learning mechanisms and psychopathology (40, 41, 52, 53).

Based on the direct involvement of dopamine in both reinforcement learning and the neurobiology of schizophrenia, more systematic experimental examinations of alterations in reinforcement learning have been reported in the last decade. With regard to aberrant salience and the described ideas about aberrant learning, so far only one experiment has been developed which specifically tests changes in adaptive (speeding up of reaction times for relevant cues) and aberrant salience (speeding up for irrelevant cues). This work by Roiser et al. (54) showed reduced adaptive salience in schizophrenia patients mostly medicated with SGAs but no general group difference in reaction time measures of aberrant salience. Within patients only, the individual degree of delusions was positively correlated with explicit measures of aberrant salience (54). Furthermore, using the same task, it was demonstrated that unmedicated people with an at-risk mental state for psychosis exhibit greater measures of aberrant salience, and this bias was correlated with their severity of delusion-like symptoms (55). Imaging results from this multimodal study (55) are reported in the next section of this article. These findings point toward the expected direction but rather support a dimensional perspective on positive symptoms, in particular delusions, in a way that the presence of aberrant learning may fluctuate with changes in clinical symptoms. Nevertheless, the findings require further validation in unmedicated patients, since antipsychotic medication directly affects dopamine neurotransmission and primarily attenuates positive symptoms. Other evidence for aberrant learning primarily comes from classical conditioning during fMRI and is reported in the next section on fMRI studies.

Studies from Gold and colleagues have contributed an important body of work to the field. These studies provide evidence for the second hypothesis that postulates a deficit in value representation (11). With regard to hedonic experience, they demonstrated that stable-medicated, chronic patients do not differ in ratings on affective picture material nor do they differ in terms of speeded motor responses to repeat or to endure viewing of these pictures. It was observed that patients respond slightly faster to repeat viewing of neutral pictures (56). These results are in line with behavioral ratings in other studies using similar affective pictures (30, 57, 58).

Together, these findings indicate that schizophrenia patients are surprisingly unimpaired in short hedonic experiences. It is important to ask how these experiences are used to learn values that may guide behavior. Studies showed that delay discounting is altered in schizophrenia in such a way that immediate rewards are preferred over larger rewards in the future and with the degree of this difference being associated with working memory deficits (59–62). A study by Heerey et al. (63) found that in two separate tasks stable-medicated, chronic patients show intact reward sensitivity but impaired weighing of potential outcomes in a decision making task: only potential losses were weighed less by patients (63). Again, the ability to use potential outcomes to guide behavior was correlated with working memory function in patients.

Hypothetically, this deficit may be based on a shift from a goal-directed to a more inflexible learning system. Even in relatively simple tasks learning speed may increase based on additional use of a goal-directed system that accurately maps separate stimulus values to their potential outcome consequences, which may then be used for appropriate action selection. Models of reinforcement learning do not map perfectly on this distinction. Instead, several agents that update values based on prediction errors can be summarized as model-free controllers of learning and decision processes, because they neglect the contribution of additional environmental features (task structure) to the learning process (compare Box 1). Nevertheless, the kind of teaching signal used to update values can even be varied within the group of model-free agents. Formally, one class includes model-free Q-learning algorithms, where each possible action becomes associated with a single value and these specific values are used to compute a prediction error. In contrast, a more rigid model-free system may learn values based on teaching signals that convey information about rewarded or punished states (e.g., a pair of stimuli) as, for example, formulated in actor-critic learning (42). This appears to be accompanied by slower learning compared to the more precise mapping of one Q-value to each stimulus associated with a certain value. Gold et al. (64) approached this question by applying a task that requires learning from rewards in one condition and the avoidance of punishment in another condition in a sample of 47 stable-medicated, chronic patients. Patients were split into two subgroups with high and low levels of negative symptoms, respectively. Only patients with high levels of negative symptoms were shown to be selectively impaired in the reward-approach condition but demonstrated intact loss avoidance learning. This dissociation was also confirmed in a post-acquisition transfer test (64). A deficit in reward-based learning, but not in the avoidance of punishment, which was associated with negative symptoms, was also found in two other independent studies, both in patients treated with antipsychotic medication (65, 66). In the study by Gold et al. (64), an actor-critic model, a Q-learner, and a hybrid of these two models were fitted to the observed data and parameters were inferred using maximum-likelihood estimation. Based on model selection, data of the high-negative-symptom group was better explained by the actor-critic model, while healthy participants and the low-negative-symptom group of patients were better explained by the Q-learner. Such a deficit in value-based learning may also be closely connected to a deficit in cost computation of effortful behavior (67). The impact of this shift to a more rigid and rather imprecise learning system may depend on task demands and may in some rare cases be advantageous – if tasks require participants to behave rigid and at low levels of exploration (68). Again, it is important to note that most of the summarized studies were conducted in stable-medicated, rather chronic patients. The important question as to what extent these findings generalize remains to be examined.

The deficit of using outcomes to guide behavior may exacerbate when patients are confronted with situations where they are required to adapt their behavior flexibly. This can be examined in tasks like the Wisconsin Card Sorting Task or reversal learning. Indeed, a deficit in such tasks has been reported repeatedly in chronic, medicated states of schizophrenia (69–73). Studies in medication-free, first-episode patients indicate that such impairments are already present at the beginning of the disease and are stable over time (for at least 6 years), independent of general IQ effects (74, 75). Two recent studies demonstrate that the deficit in rapid behavioral adaptation is most likely due to an increased tendency to switch in schizophrenia patients (76, 77). A study by Schlagenhauf et al. (77) implemented detailed computational modeling of learning – ranging from standard Rescorla–Wagner-Models to Double-Update-Models (Box 1) and finally belief-based Hidden–Markov-Models (78) – to the data of 24 unmedicated patients. While the used Rescorla–Wagner-Models clearly provide a model-free account of reinforcement learning, the Double-Update- and the Hidden–Markov-Models can both be regarded as a model-based account of reinforcement learning because both incorporate important aspects of the experimental environment of the given task but in different ways: the Double-Update-Model simply integrates the dichotomy of the two choice options in the reversal learning task by updating each action value with the same prediction error but in different directions; the Hidden–Markov-Model approaches this differently by updating the probability of being in one of the two states and thereby actually building an internal model of the task’s states (in the following, this is referred to as the participant’s belief about the visited trial being informative about the state or not). Maximum-a-posteriori estimates of model parameters were inferred using random-effects Bayesian techniques complemented by model selection at the population and at the individual level. Random-effects parameters refer to individual parameter estimates per participant in contrast to fixed-effects parameters, which assume one set of parameters for a population. Note that random-effects fitting of models and model selection are crucially important to compare how models map to learning processes across groups and to compare parameters between groups. Also, individual model comparison is important because the meaning of underlying parameters remains unclear if the probability that a participant’s data is given by the inferred parameters (the likelihood) is around chance (please also compare Section “Methodological Remarks”). Based on these methods, it was demonstrated that the belief-based model explained the observed data best. This is in line with another study on reversal learning in healthy participants (78). Modeling results revealed increased switching in patients due to false beliefs with respect to feedback-conveyed information about the state of the task, which are based on reversals of reward contingencies (77). The study by Schlagenhauf et al. (77) was conducted in 24 unmedicated patients, of whom a substantial number was not able to apply the belief-based strategy. In these patients (*n* = 11), the reversal learning deficit was more pronounced. This was best explained by the actual presence of their positive symptoms, which is a remarkable contrast to several studies examining stable-medicated, chronic patients with attenuated positive symptoms. This subgroup of patients was additionally characterized by the model in terms of reduced reward sensitivity and showed a relatively better (although still poor) fit by the simple, model-free Rescorla–Wagner algorithm. Parameters of the models were used to generate regressors for the analysis of fMRI data and the results are discussed in the subsequent section.

There is convincing support that deficits in flexible behavioral adaptation and reversal learning, in particular, are important features of schizophrenia patients with an increased tendency to switch as a potential specific mechanism (76, 77). This is in line with an important assumption concerning the hypothesis of a deficit in value representation: an impaired functioning of the so-called rapid learning system that is assumed to rely on prefrontal and orbitofrontal brain structures deeply involved in cognitive functions such as working memory, which allows for flexible adaptation of decisions (47). This system is thought to interact with a more rigid learning system supposedly implemented in the basal ganglia pathways. As already mentioned above, these complementary learning systems may also be associated with the distinction of model-free and model-based controllers of learning, where the latter is implicated in using an internal model of the environment to optimize choice behavior (79, 80). It appears plausible that potential deficits in the model-based domain may be closely linked to well-established findings of impaired cognitive control with most evidence from measures of working memory and cognitive processing speed. Model-based learning relies on precise mapping of the environment and uses this map for forward planning of decisions. This process requires individuals to keep online values of multiple stimuli to allow for flexible decision making.

There is indeed evidence that working memory capacity limits the ability to learn multiple stimulus values to guide decisions and the degree of model-based behavior (81, 82), while, at the same time, possibly directing patients toward more inflexible aspects of learning, which themselves may be affected or spared in schizophrenia. There is additional evidence that patients learn reward contingencies, but that they may need more time depending on task demands (68, 83, 84). Interestingly, in a post-acquisition test-phase, Waltz et al. (83) observed that medicated patients learned to avoid previously punished stimuli, while preference for the previously rewarded cues was weakened compared to controls. In a next step, Waltz et al. (85) studied stable-medicated, chronic patients with an established go-nogo learning task (86). During the training phase, patients showed an overall go-bias but no gradual adaptation to the more frequently rewarded stimuli, while the gradual adaptation to negative outcomes appeared to be intact (85). In line with deficits in reversal learning, rapid trial-to-trial adjustments were impaired in patients. This analysis was compared with predictions from a neurocomputational model of dopamine-induced basal ganglia-cortex interactions proposed by Frank et al. (87): high levels of presynaptic dopamine accompanied by alterations in D1-receptor density may specifically impair go-pathways which are proposed to facilitate reward-approach rather than punishment avoidance (47). This idea is also supported by recent optogenetic animal research (88). In accordance, it was also demonstrated that patients are less able to speed up responses to approach reward and show reduced exploration. Both effects were most pronounced in a subgroup of high-level negative symptoms (89).

In this section, we summarized results from studies on behavioral impairments during performance of instrumental tasks and only three studies, to date, have implemented reinforcement learning modeling to the observed behavioral data (64, 77, 89). Two of those studies demonstrated the ability to identify subgroups of the heterogeneous clinical entity referred to as schizophrenia. Further studies with similar experiments are needed across different disease states (e.g., first-episode) and medication states (in particular unmedicated patients as well as different medications to rule out the possibility that alterations in learning mechanisms are secondary to medication effects). This may be a potentially helpful route toward an identification of patient subgroups based on generative computational models of behavior and neural mechanisms. Recent methodological progress shows improved classification accuracy and allows for clustering within patients based on parameters of generative models of brain connectivity (90, 91), and this may also apply to generative models of behavior.

## Functional Imaging Studies of Reinforcement Learning in Schizophrenia

This section will summarize studies that collected fMRI data during reinforcement learning to examine neural substrates of the behavioral alterations discussed in the previous section of this article. First, we summarize studies that examined classical conditioning. This process of associative learning has not been discussed in the previous section because classical conditioning paradigms do not usually require an instrumental response. Nevertheless, physiological responses reflect associative changes in stimulus contingencies, namely the unconditioned and the conditioned stimuli (US and CS). Second, we report studies that investigated instrumental conditioning during fMRI. In both parts, we explicitly describe the application of reinforcement learning models, how parameters underlying these models were inferred, and how these measures were further applied to the imaging data.

### Classical conditioning

Jensen et al. (92) studied aversive classical conditioning in 13 medicated patients. Their analysis focused on the onset of CS associated with a neutral or an aversive event. In patients, they found elevated left ventral striatal activation to CS preceding neutral events compared to CS preceding aversive events (92). This aberrant attribution of salience was confirmed in skin conductance measures and post-learning self-reports. In a slightly different aversive conditioning paradigm neural responses to CS and US were studied in 20 medicated patients, and similar findings were demonstrated (93): attenuated activation to CS but intact responses to US were reported in the amygdala. Within patients, CS-related activation in the midbrain was correlated with delusion severity in a way that stronger CS-related responses in neutral trials predicted a higher degree of delusional symptoms (93). The authors additionally implemented a temporal-difference model to quantify neural correlates of prediction errors. Notably, the model’s free parameter, the learning rate, was fixed for the entire sample and not fitted individually to behavioral or physiological responses [which were shown to vary, according reaction times and skin conductance e.g., Ref. (94, 95)]. Romaniuk and colleagues found no aversive prediction error correlate in the midbrain of schizophrenia patients as was observed in healthy controls. When modeling prediction errors for neutral events, they found a neural correlate of these prediction errors in patients’ midbrain but not in controls (93).

With regard to appetitive classical conditioning with monetary reward, one study investigated neural activation to reward-associated CS in 25 medicated patients. They reported that relatively lower ventral-striatal and ventro-medial-prefrontal activation depended on the degree of anhedonia (96), which is in line with previous findings using the MID task (17). Another study examined appetitive classical conditioning in thirsty participants (15 medicated patients) using water as reward. The analysis focused on reward delivery and found blunted ventral striatal activation in patients to be correlated with negative symptoms (97). Further, functional connectivity of the dopaminergic midbrain with the insula was reduced in patients. Another appetitive classical conditioning paradigm with monetary reward was used in a study by Diaconescu et al. (98) in 18 medicated patients. While patients and controls were similarly able to recall reward contingencies in explicit ratings, implicit measures (skin conductance) did not differ between reward CS and neutral CS in patients. The analysis of fMRI data also focused on CS and revealed that increased activation in striatal and prefrontal areas of healthy controls to reward CS was accompanied by stronger effective connectivity between VS and orbitofrontal cortex as assessed using structural equation modeling (98). Crucially, this pattern was reversed in patients for the neutral CS. This is an important finding, as it has long been described that neural correlates of learning spread over nodes of a network and thereby drive changes in plasticity. A disturbance of such a mechanism was also proposed to be at the heart of the pathophysiology of schizophrenia (99–101). We will return to this issue in the final section.

### Instrumental learning

We now proceed with further studies that investigated neural correlates during instrumental learning. In line with evidence for aberrant learning from classical conditioning, a recent multimodal imaging study using the instrumental “salience attribution task” [(55); for behavioral results see previous section] found that ventral striatal activation to irrelevant stimulus features were positively correlated to delusion-like symptom severity in 18 unmedicated people with an at-risk mental state for psychosis (55). Furthermore, hippocampal responses to irrelevant features were differently correlated with dopamine synthesis capacity in VS revealing a positive relationship in controls and a negative relationship in people with an at-risk mental state.

One exemplary study that assessed the association between impaired reinforcement learning and brain activation in dopaminergic target brain areas of first-episode schizophrenia patients (*n* = 13, 8 medicated) used an instrumental learning task with two choice options: one signaled a potential monetary feedback and the other a potential neutral feedback (43). In contrast to several other studies (see previous section), the groups did not differ in terms of acquisition of reward contingencies, which may be due to the rather small sample size of this pioneer study. In line with another study (59), patients responded faster on neutral trials in the study by Murray et al. (43). A Q-learner was fitted to the observed data based on maximum-likelihood estimates of parameters. Both groups did not differ in terms of model parameters. To generate regressors for fMRI data analysis, one set of parameters was fitted for the entire sample (fixed-effects). Model-derived prediction errors were used as a parametric modulator of feedback events. Prediction error correlates in bilateral midbrain, right VS, hippocampus, insula, and cingulate cortex were significantly stronger in controls than in patients. In patients, midbrain correlates of prediction errors appeared slightly augmented in neutral trials (43). A more complicated “allergy prediction” task design enabled Corlett et al. (102) to investigate different stages of learning in 14 patients, most of whom were medicated. For event-related fMRI analysis, an event was defined to start at the beginning of each stimulus presentation and to end after outcome delivery lasting a total time of 4 s. Compared to controls, patients did not activate the left caudate during the training stage, which was followed by revaluation of stimuli pairs that were either ambiguous or well learned pairs of cues during training. The comparison of these pairs revealed a failure to activate substantia nigra and right PFC. In the last phase, expectations about the outcome based on the trained stimulus pairs were violated. Here, predictable events elicited an augmented response in right PFC in patients versus controls, while an attenuated response was found for unexpected events (102). This lack of differentiation between expected- and unexpectedness events correlated with the level of unusual thought content. Notably, the analysis strategy chosen in this design makes it hard to interpret the findings in terms of prediction error or expected value signals because the whole trial period was modeled in the single-subject of the fMRI data. Similar results were reported in another study that investigated 20 medicated patients while performing a guessing–gambling paradigm at different levels of uncertainty but analyzed expectation-related and reward-related activation separately (103). Expectation-related brain activation at time of motor responses revealed increased activation with lower predictability in a fronto-parietal network, and this effect was diminished in dorsolateral PFC and anterior cingulate cortex of schizophrenia patients. Reward-associated activation was analyzed in relation to levels of predictability (assumed to mirror prediction error related brain activation), and patients showed reduced activation in putamen, dorsal cingulate, and superior frontal cortex (104). One study assessed probabilistic category learning (“weather prediction task”) in medicated schizophrenia patients (*n* = 40) during fMRI. Albeit impaired performance in all patients, a small number of patients were able to apply a similar strategy to the task as controls did (105). When comparing fMRI data of these matched groups (*n* = 8 patients) during the presentation of stimulus combinations, patients displayed reduced activation in striatum and dorsolateral PFC. Patients exhibited stronger activation in a more rostral region of dlPFC and parietal cortex. Results from this task are hard to compare with instrumental reinforcement learning tasks due to the experimental design that primarily tests classification learning at different levels of difficulty.

In another study on instrumental learning, Gradin et al. (106) examined 15 medicated patients. Temporal-difference modeling was applied to the task that delivered water as reward. Random-effects parameters were initially estimated with maximum-likelihood, and the obtained parameters were subsequently used as empirical priors to regularize the possible range parameters to avoid extreme values of parameter estimates [also compare: Ref. (53, 106)]. Although patients differed in the amount of delivered water, no difference on model parameters was observed. To generate regressors for fMRI analysis, a single set of parameters was fitted for the entire sample (fixed-effects). Model-derived prediction errors were analyzed as parametric modulators of reward delivery, and model-derived values were included as modulators of expectation-related activation at the trial onset. Compared to controls, no correlation with prediction errors was observed in striatum, thalamus, amygdala-hippocampal complex, and insula of medicated schizophrenia patients. A trend-wise reduction in midbrain correlated with positive symptoms in patients. Patients also displayed reduced coding of value-related activation in the amygdala-hippocampal complex and this, again, was correlated with positive symptoms. Importantly, this study also included another psychiatric patient group, medicated depressed patients, and this group also exhibited blunted neural correlates of expected-reward values and prediction errors in slightly different regions. The strength of this reduction was correlated with anhedonia severity in dopaminergic core areas. In combination with detailed computational modeling, Schlagenhauf et al. (77) studied reversal learning (compare previous section) in 24 unmedicated patients. Analysis of fMRI focused on the time of reward delivery and included different model-derived modulations of this onset. The authors found reduced ventral striatal coding of model-derived reward prediction errors in patients. This finding remained trend-wise significant when restricting the group comparison to patients who had insight into the underlying task structure as defined by their beliefs about the states of the task based on a Hidden–Markov-Model (*n* = 12). A second fMRI analysis based on the latter model was applied to define subjective informative punishment trials, i.e., when participants believed that a change in reward contingencies had appeared. Both patients with good and poor task insight showed reduced ventral striatal activation during these trials (77). Reduced ventral striatal activation was also reported in another recent fMRI study on reversal learning in 28 medicated, chronic schizophrenia patients (76). In the study by Schlagenhauf et al. (77), patients with good task insight displayed relatively stronger activation of ventro-lateral and dorso-medial PFC than patients with poor insight. Well performing patients were not distinguishable from controls with respect to activation in these prefrontal regions. This result may reflect compensatory PFC processes in schizophrenia patients similar to that which has been described for the neural correlates of working memory deficits (107, 108).

In summary, several studies revealed reduced activation of brain areas typically encoding errors of reward prediction, most prominently the VS. This was reported consistently across classical and instrumental conditioning tasks, despite the fact that most of these studies differ enormously with regard to experimental designs and analysis strategies. Prediction errors arise when a reward is delivered and are typically thought to train expected values of stimuli or associated actions (42). Therefore, functional neuroimaging studies that studied learning during scanning have so far helped to elucidate the underlying dynamics of previous findings derived from studies using the MID or similar tasks. That is, neuronal teaching signals are not coded in ventral striatal activation of medicated and unmedicated patients to a similar extent as in controls. Only five imaging studies have applied reinforcement learning models to describe this process on a trial-by-trial level and these vary considerably in terms of the implemented models, inference of model parameters and the application of model-derived measures to the imaging data. We will further comment on these issues in the subsequent section. These studies comprised 78 medicated patients and 24 unmedicated patients. Studies in unmedicated patients are still rare. Nevertheless, the finding of reduced prediction error coding in dopaminergic core areas may indeed build a common ground for impaired learning of stimulus or decision values. In addition, such impaired coding might be closely related to the elevated levels of presynaptic dopamine synthesis capacity in schizophrenia reported in meta-analyses of PET studies (4, 5, 109). An important question remains how this stable marker of the dopamine system, probably reflecting tonic or rather stable aspects of dopaminergic neurotransmission (3), relates to event-related changes during learning. Studies approaching this question are discussed in Section “Functional Imaging Studies of Reinforcement Learning with Additional Neurochemical Measures or Pharmacological Challenges of the Dopamine System” of this article. Furthermore, it has been proposed that a hyperdopaminergic state in schizophrenia may result in imprecise and inefficient cortical information processing as a potential mechanism for cognitive impairments observed in patients as well as their first-degree relatives and in people at-risk mental states (9, 110, 111). This idea is compatible with the proposal of a deficit in prefrontal value representation shown to be related to negative symptoms. However, exact cognitive and affective correlates of such deficits remain to be explored. We will return to this in the final section.

The emerging picture is less clear with regard to evidence provided in favor of the aberrant salience hypothesis, in particular regarding the extent to which reduced neural correlates of prediction errors are linked to processes of aberrant salience attribution. Notably, the idea of aberrant salience may also account for reduced value-related anticipatory dopaminergic signals, in patients who exhibit high levels of positive and negative symptoms (for example). In this case, a lack of activation to cues associated with monetary as well as, probably, social reward may reflect reduced motivational or incentive salience in terms of apathy or other dimensions of negative symptoms, which may be a result of aberrant salience attribution. However, this requires more systematic studies along symptom dimensions. Evidence for neural correlates of aberrant learning was demonstrated in fMRI studies on classical conditioning that showed elevated striatal activation to cues indicating the delivery of a neutral event (92, 93, 98) and in one specific instrumental task design, the “salience attribution task” (55, 112). Studies using this specifically designed task point toward a relationship with positive symptoms, particularly delusions. Consequently, symptom and medication states of included patients may be crucially important. Indeed, a study on reversal learning in unmedicated patients with more pronounced positive symptoms showed that a subgroup of patients was not able to infer the task structure and this was best explained by individual levels of positive symptoms (77). Therefore, it is important to consider the amount of variance in symptom ratings and different medication states to better understand variability related to aberrant aspects of neural learning signals. Furthermore, when reviewing clinical data of several studies summarized in this article, it is compelling that even in medicated patients there is considerable variability in the extent of positive symptoms across studies varying from high levels to nearly no positive symptoms. Future studies are needed to address the question whether blunted learning signals indeed reflect aberrant salience attribution – and if this is a schizophrenia specific feature or a dimension of positive psychotic symptoms – which may then consequently also emerge in other psychiatric diseases and to some extent even in the at-risk healthy population or healthy people with some degree of psychotic experience.

## Methodological Remarks

The combination of model-derived learning signals with functional brain measures is very promising. This mechanistically informed quantification of signals reflecting learning processes provides a more fine-grained insight into neural trial-by-trial correlates of learning mechanisms and disease-specific alterations as compared to standard event-related fMRI analyses which rather rely on event definitions such as correct responses or experimenter-defined changes in reward contingencies. In fact, the latter may not always reflect the way study participants solve these tasks. On the other hand, a small number of healthy volunteers, in most studies, exhibit behavior that cannot be described better than chance by any reinforcement learning model. This may indicate the need to extend from standard reinforcement learning models to other types of models, for example Bayesian learners (94, 113, 114). Such non-fitters should be reported more clearly, in particular in clinical between-group studies, because this may crucially impair the between-group analysis of model parameters and comparisons of neural correlates based on model-derived measures between groups: in fact, underlying parameters of non-fitters are meaningless in terms of the mechanism that is described by the model [compare Ref. (77)]. Although studies which actually apply reinforcement learning modeling are the minority of those reported in this review article (seven studies, for an overview see Table 1), there is considerable variability on how these few studies inferred the models’ parameters (some did and others did not fit parameters) and how (or if any) model selection was applied.

Further, the generation of trial-by-trial model-derived time-series for fMRI data analysis is sometimes performed based on random-effects parameters (individual parameters for each subject) or based on one set of parameters (fixed-effects). One group recommends the latter approach for studies in healthy volunteers by arguing for more robust correlations of BOLD signal with model-derived regressors (53). On the other hand, this appears questionable for group studies in which group differences in parameters may be causally linked to the disease status. We have the impression that model comparison techniques are of key importance (115). Even in the simple case that no alternative models are fitted, it may be informative to include a report of model fit based on the likelihood that the observed data is given by the parameters. To our mind, a situation where the individual model fit (expressed via the likelihood of the data given by the parameters) does not differ between groups exemplifies a desirable case: even if parameters differ between groups in this case, model-derived regressors are readily applicable to fMRI data because they do not differ in terms of the likelihood that the modeled strategy captures important aspects of the observed raw responses. Based on the sparsely available papers on these issues, the application of fixed-effects parameters to fMRI data rather appears as a workaround based on the observation that noisy parameters based on maximum-likelihood estimates potentially add further noise when fitting a hemodynamic model with model-derived time-series as parametric modulators to the imaging data [compare Ref. (53)]. In the case of clinical between-group studies, the use of fixed-effects parameters results in a situation where the observed behavior is relatively well explained by those parameters. Consequently, differences in terms of model parameters will then be expressed via the correlation between the regressor and the signal. This can be minimized by using parameters that closely match the observed individual’s behavior to generate regressors. Unfortunately, no systematic studies of these questions are available involving either healthy volunteers only, or comparisons between psychiatric patients and healthy controls. Consequently, it appears to be desirable to develop methodological guidelines for these techniques, as it was published for other modeling approaches, for example for dynamic causal modeling for fMRI (116).

## Functional Imaging Studies of Reinforcement Learning with Additional Neurochemical Measures or Pharmacological Challenges of the Dopamine System

In this last section, we describe research that pharmacologically manipulated the dopamine system during reinforcement learning or acquired an additional molecular measurement (PET) of dopamine. There are a substantial number of groups researching these questions in healthy volunteers, and this section does not aim to present a complete picture of all such studies. We will instead refer to studies that are particularly important for a better understanding of the above reviewed studies in patients.

A highly influential study was conducted by Pessiglione et al. (117). An instrumental learning task with a reward-approach (win or no win) and a punishment–avoidance (loss or no loss) condition was used (117). A similar design was recently applied in a behavioral study of medicated schizophrenia patients (64). Pessiglione et al. (117) demonstrated that dopamine medication, l-DOPA, and haloperidol, have opposing effects on behavior and neural correlates of model-derived prediction errors, and that these effects are selective for the reward-approach condition: l-DOPA administration enhanced reward-approaching behavior and associated ventral striatal reward prediction errors whereas haloperidol reduced such effects (117). The same direction of medication effects was reported in a study using aversive Pavlovian conditioning under amphetamine, haloperidol, or placebo (118). In addition, ventral striatal reward anticipation as in the MID task appears to be conveyed by reward-induced dopamine release (27) and can be blunted by massive dopamine release, based on dose-dependent effects of psychostimulants (26, 28). In line with this, a recent study applied the same reward-approach task as in Murray et al. (43) and found that methamphetamine blunts both reward prediction errors in VS and expected value representation in ventro-medial PFC (119). The strength of the disruption of value representation in ventro-medial PFC was correlated with amphetamine-induced psychotic symptoms. A third condition, amisulpride-pretreatment, did not affect amphetamine-induced blunting of learning signals. It is important to note that the reducing effects of haloperidol on striatal reward prediction error encoding can explain reduced prediction error related activation in medicated schizophrenia patients, whereas the blunting effects of amphetamine may potentially mirror a subcortical hyperdopaminergic state, as was demonstrated in unmedicated schizophrenia patients (2, 3). Therefore studies in unmedicated patients are crucially important to remove this confound. FGAs and SGAs strongly differ in their dopamine receptor affinity, and, based on two MID studies, it was shown that they also affect reward anticipation differently. These results point toward the idea that SGAs may help to remediate reward-related anticipatory brain activation (20–22) which nevertheless requires random assignment in a clinical-trial-type design. Such studies have not yet been conducted with learning tasks. In unmedicated patients, a reduction of ventral-striatal prediction error coding was recently demonstrated during reversal learning (77). Elevated presynaptic dopamine levels may account for this reduced activation, similar to that observed for Parkinson patients on l-DOPA medication, affecting the VS (early in the illness less degenerated) in an overdosing manner (120). Here, a long-lasting increase of presynaptic dopamine function may “drown” value-related and error-related phasic learning signals. Multimodal imaging studies combining fMRI with PET radiotracers that assess presynaptic dopamine function can link individual differences in neurochemical parameters with functional activation. For example, PET with FDOPA may be an important target for application in multimodal imaging studies, since this measure has been demonstrated in meta-analyses to best characterize the subcortical hyperdopaminergic state of patients [for meta-analyses see: Ref. (4, 5)]. Supporting this idea, another study demonstrated that ventral-striatal prediction errors are indeed negatively correlated with dopamine synthesis capacity in healthy controls (121). This negative correlation suggests that long-lasting increases in presynaptic dopamine function, as observed in schizophrenia patients, may reduce phasic learning signals, hypothetically via presynaptic D2-autoreceptors which regulate presynaptic activity of DOPA-decarboxylase activity to ensure homeostasis within the dopaminergic system (46, 121). Animal studies (122, 123) and other functional human imaging studies (36, 124, 125) also support the idea of this interplay of differential aspects of dopamine neurotransmission. In line with this, it has also been shown that behavioral effects of a dopamine-enhancing drug during reversal learning indeed depend on baseline levels of dopamine synthesis capacity (126): Participants with lower dopamine synthesis capacity benefit behaviorally from a dopamine agonist, while the same drug dose seemed to be disadvantageous for participants with rather high levels of dopamine synthesis capacity. Therefore, dopamine effects in learning and cognition appear to be a fine-tuned and optimized non-linear system where rather low and rather high levels result in inefficient neural processing (127, 128). This view is also supported by one of the few clinical multimodal imaging studies using FDOPA PET in combination with a working memory task during fMRI in controls and in people with an at-risk mental state for psychosis (129). At the same working memory load, they found a positive linear relationship of dopamine synthesis capacity and working memory related activation in dorsolateral PFC of healthy controls, while this relationship was negative in people with an at-risk mental state, indicating that potentially “too” high levels of dopamine synthesis may promote lower dorsolateral PFC activation during the same working memory load at which both groups coped with comparable performance. This observation can be reconciled with the observation of prefrontal efficiency during working memory when examining cognitive performance and dorsolateral PFC activation (130, 131): different dorsolateral PFC activation may primarily reflect different performance. Patients are assumed to reach maximum limits of dorsolateral PFC activation earlier reflecting a general impairment in this cognitive domain [see also: Ref. (108)]. A step further, there is also evidence that a reduction of working-memory-dependent effective connectivity from dorsolateral PFC to parietal cortex may be the potential mechanism underlying this inefficiency (108). Connectivity may indeed be an important target and it has also been demonstrated that functional connectivity during aversive conditioning is shifted differently by a dopamine agonist versus a dopamine antagonist (132).

## Conclusion, Remarks, Outlook

In this review article we summarized studies that provide evidence for behavioral and neural correlates of impaired reinforcement learning in schizophrenia. Two main hypotheses guided this review: (1) Aberrant prediction errors drive learning of otherwise irrelevant stimuli and actions in schizophrenia, and that there is a potentially close link between this mechanism and the emergence of positive psychotic symptoms, in particular delusions. (2) A deficit of expected value representation may characterize patients suffering from schizophrenia, and this may fundamentally contribute to the formation of negative symptoms.

There is evidence for aberrant learning with most specific findings derived from the salience attribution task (54, 55). Although there is still limited evidence and future studies are needed for clarification, it seems conceivable that aberrant learning is involved in the formation of delusions and can therefore be observed in patients with prominent positive symptoms. Whether this sensitization to otherwise neutral stimuli is indeed dopamine mediated and actually blunts learning signals elicited by regularly salient cues remains to be further explored (9).

Our summary of fMRI studies during reinforcement learning clearly demonstrates that a reduction of these learning signals, namely blunted coding of ventral-striatal prediction errors, was consistently observed across studies. This deficit may actually be involved in aberrant learning as well as in a failure of value representation depending on fluctuating symptom states. During acute psychotic clinical states this may provide a conduit for aberrant learning, while the persistence of a noisy learning signals may provide the ground for a failure of building value expectations, ultimately contributing to the development and progress of detrimental negative symptoms. A large body of evidence from behavioral studies supports the hypothesis of a deficit in value representation and that this impairment is pronounced in patients with high levels of negative symptoms (11, 64). Nevertheless, antipsychotic medication was shown to contribute to the severity of negative symptoms based on the degree of striatal D2-receptor blockade (133) and may therefore also exacerbate impairments in value representation.

The psychosis spectrum has been characterized by imprecise and inefficient cortical information processing as a potential mechanism for cognitive impairments observed in patients and their first-degree relatives as well as in at-risk mental states. As a potential mechanism behind this, a disrupted cortico-cortical synaptic plasticity was suggested by a comprehensive biological hypothesis of schizophrenia, the “dysconnectivity” hypothesis (100, 101). This hypothesis focuses on aberrant experience-driven control of synaptic plasticity via *N*-methyl-d-aspartate receptors (NMDAR). Abnormal modulation of NMDAR-induced plasticity by neurotransmitter systems such as dopamine, acetylcholine, or serotonin are at the heart of this idea. In the present article, we have described a close link between reinforcement learning, symptom dimensions of schizophrenia and dopamine, which acts as a neuromodulator of NMDAR-function: Animal research demonstrated that D1-receptor agonists and D2-receptor antagonists facilitate NMDAR-dependent long-term plasticity while D2-receptor agonist suppress it (134, 135). Earlier in the manuscript, we have discussed the role of these receptors during reward and punishment as well as go-nogo learning (87, 88). Further, these receptors are targets of current antipsychotic treatment strategies. Stephan and colleagues conclude “… it is not plasticity *per se* that is abnormal but its modulation during reinforcement and perceptual learning.” (100) These modulatory influences of NMDAR-function are thought to contribute to cortical representations of environmental states (136) and the consistently described reduction of ventral-striatal prediction errors could be crucially involved in a deficient shaping of such cortical representations (137, 138). Here, it is important to underline that aberrant neuromodulation can indeed be formulated via computational models of learning-induced plasticity.

So far, there is converging evidence that dysconnectivity may indeed account for the repeatedly described prefrontal inefficiency observed in schizophrenia during the performance of cognitive tasks: Using models of effective connectivity for fMRI, reduced working-memory-dependent prefrontal-parietal connectivity was reported, initially in medicated patients (108) and subsequently replicated in medication-naïve first-episode patients and in people with an at-risk mental state (139). Based on parameters of these models, a clustering analysis was able to identify three mechanistically informed subgroups of patients (91). These subgroups were found to be biologically distinct in terms of connectivity profiles and mapped on different levels of negative symptom severity (91). This observation also appears to be in accordance with the proposal of a deficit in value representation, which was demonstrated to be pronounced at high levels of negative symptoms (64). Therefore, it appears desirable to study the effect of neural learning signals at various levels in neural networks. It is important to note that studying the interaction of model-free learning signals and model-based neural representations of cognitive processes on the level of neural networks in a computational framework clearly has the potential to move beyond the evidence provided by standard cognitive tasks, such as working memory, even if some of the identified deficits overlap. The contribution of such an approach can be to gain more mechanistic information when studying these processes by applying detailed computational modeling to behavioral and neurobiological data. The focus of this idea is that different types of computational processes described in terms of different models may help us to improve our understanding of how patients actually solve certain tasks beyond the observation of being impaired or not. This may offer a unique source for mechanistically informed subtyping based on how patient subgroups deal differently with challenging tasks and in particular how these abilities are implemented in neural networks. Such subgroups require clinical validation in terms of longitudinal predictions (e.g., treatment responses). Promising future research in this field may strongly benefit from an integration of different modeling techniques for reinforcement as well as perceptual learning and brain connectivity [e.g., dynamic causal modeling; (140)]. It has been demonstrated in healthy volunteers that such an experimental approach is feasible (94, 141, 142) and therefore presents a highly promising venue for schizophrenia research. Finally, this may result in a dissection of the heterogeneous clinical entity of schizophrenia into biologically informed subgroups, thereby providing a framework for a better understanding of cognitive deficits, where a deficit of learning expectations about sensory inputs and future actions may constitute a potential key mechanism of the disorder.

## Conflict of Interest Statement

The authors declare that the research was conducted in the absence of any commercial or financial relationships that could be construed as a potential conflict of interest.
